# Enigma of IL-17 and Th17 Cells in Rheumatoid Arthritis and in Autoimmune Animal Models of Arthritis

**DOI:** 10.1155/2016/6145810

**Published:** 2016-01-20

**Authors:** Reka Kugyelka, Zoltan Kohl, Katalin Olasz, Katalin Mikecz, Tibor A. Rauch, Tibor T. Glant, Ferenc Boldizsar

**Affiliations:** ^1^Department of Immunology and Biotechnology, University of Pécs, Szigeti ut 12, Pécs 7624, Hungary; ^2^Section of Molecular Medicine, Rush University Medical Center, 1735 West Harrison Street, Chicago, IL 60612, USA

## Abstract

Rheumatoid arthritis (RA) is one of the most common autoimmune disorders characterized by the chronic and progressive inflammation of various organs, most notably the synovia of joints leading to joint destruction, a shorter life expectancy, and reduced quality of life. Although we have substantial information about the pathophysiology of the disease with various groups of immune cells and soluble mediators identified to participate in the pathogenesis, several aspects of the altered immune functions and regulation in RA remain controversial. Animal models are especially useful in such scenarios. Recently research focused on IL-17 and IL-17 producing cells in various inflammatory diseases such as in RA and in different rodent models of RA. These studies provided occasionally contradictory results with IL-17 being more prominent in some of the models than in others; the findings of such experimental setups were sometimes inconclusive compared to the human data. The aim of this review is to summarize briefly the recent advancements on the role of IL-17, particularly in the different rodent models of RA.

## 1. Introduction

Rheumatoid arthritis (RA) is a systemic autoimmune disorder characterized by chronic synovitis leading to the progressive destruction of joints accompanied by systemic inflammation and the production of autoantibodies [1]. RA affects 0.5–1 percent of the human population making it one of the most common autoimmune disorders. Since the first modern description of the disease in 1800 [2], our knowledge regarding the pathomechanisms of RA has expanded to such a degree that specific therapies targeting various modulators of the inflammatory phase could be introduced, truly revolutionizing the treatment of RA. Yet little is known about how and when the disease starts although the new therapeutic agents proved to be much more effective than the conventional drugs used earlier. However, these disease-modifying antirheumatic drugs (DMARDs) do not alter the autoimmunity per se; they do not lead to remission in all of the patients. The central role of TNF*α* in the inflammatory phase of RA has been described earlier, but with the description of Th17 cells and the involvement of IL-17 in RA some new potential treatment options became available only recently [3]. Our review will mainly focus on the role of IL-17 in RA, particularly the rodent models of the disease, which all contributed to the development of novel therapeutic agents.

## 2. Th17 Cells in RA

The synovitis in RA is characterized by massive cellular infiltration of the synovium consisting mainly of leukocytes such as T and B cells, macrophages, granulocytes, and dendritic cells together with the increased local production of proinflammatory cytokines and chemokines, eventually leading to the destruction of the joint and bone. T cells, especially CD4^+^ T cells, play a major role in this process, also supported by the effective use of Abatacept in the treatment of RA, an agent that selectively blocks T cell costimulation [4]. For a long time RA was considered to be a Th1-dependent disease. However, following the description and characterization of IL-17 and Th17 cells, more and more data indicated that these latter types of CD4^+^ cells are key players in the development of RA and that anti-IL-17 therapies might have beneficial effects [3].

Th17 cells are a subgroup of helper T cells with the capability to produce high levels of IL-17 described as their main characteristic along with the expression of the chemokine receptor CCR6 and the transcription factor ROR*γ*t (RAR-related orphan receptor gamma t) [5]. In humans, Th17 commitment requires the production of mainly IL-1*β*, IL-6, IL-21, and IL-23, all of which are produced by tissue-resident activated macrophages and dendritic cells in an inflammatory environment [6, 7]. These members of the innate immune system are not only capable of inducing Th17 commitment but they also participate in the recruitment of Th17 cells to the site of inflammation through the production of chemokines that bind CCR6. CCR6 is expressed by a variety of cells such as immature dendritic cells, regulatory T cells (T_reg_), and Th22 and Th17 cells. An increased proportion of CCR6^+^ Th17 cells were described in the peripheral blood of patients with early untreated RA [8], and Th17 cells infiltrated the joints as these cells were detected both in the synovial fluids and in synovial membranes of RA patients [9, 10]. While the number of Th17 cells in the peripheral blood does not seem to be a reliable diagnostic tool to date, their increased presence in the synovial fluid correlates with increased disease activity in RA [11].

In addition to elevated IL-17 levels, an increased concentration of CCL20 was also detected in the synovial fluid of RA patients, and* in vitro* cultured synovial fibroblasts of RA patients were capable of producing CCL20 after treating them with IL-1 and TNF*α* [12, 13]. It was demonstrated that CCL20 acts as a chemoattractant of the CCR6 expressing Th17 cells in mice. Human peripheral blood Th17 also expressed CCR6 (see above); thus CCL20 might be a key element in the recruitment of Th17 cells to the inflamed joints of RA patients [13]. Therefore, CCL20 is the most significant ligand of CCR6, and the CCL20/CCR6 axis may serve as potential therapeutic target.

## 3. Discovery of IL-17

Interleukin-17 (IL-17), originally termed as CTLA-8, was first identified in 1993 as a transcript from a cDNA library derived from a T cell hybridoma generated by the fusion of murine cytotoxic T cells and rat T cell lymphoma cells [14]. The sequence showed 58% identity to the Herpesvirus Saimiri gene 13 (HSV13) and both the recombinant CTLA-8 and HSV13 stimulated the NF-*κβ* pathway leading to increased IL-6 production in fibroblasts and also stimulated T cell proliferation acting similarly as the proinflammatory cytokines [15, 16]. Later, the CTLA-8 was renamed IL-17 [15] and in 1996 the originally identified rat CTLA-8 was confirmed as a homologue of the murine IL-17 [17].

In 1986, murine helper T cells were divided into Th1 and Th2 subtypes based on the cytokines they produced [18]. According to this hypothesis the naive CD4^+^ T cells could differentiate either into IFN*γ*-producing Th1 or IL-4-producing Th2 cells, a process which is controlled mainly by the antigen presenting cells (APC) [19]. The discovery of IL-17 greatly challenged the former bipolar classification of helper T cells [20]. First, the amino acid sequence of IL-17 significantly differed from other cytokines previously described, and the structure of its receptor did not fit into those of other cytokine receptor families, making IL-17 a seemingly distinct signaling molecule. Subsequently, it was demonstrated that naive T cells primed with the lysate of* Borrelia burgdorferi* develop a phenotype characterized by markedly increased IL-17 production. However, IL-17 could not be classified as a Th1 or Th2 cytokine, which led to the discovery of Th17 cells as a distinct CD4^+^ T cell population [21]. Since then, a large number of studies have been performed to reveal the physiological role of IL-17 and Th17 cells, as well as their participation in pathological conditions. One of the most targeted areas was the autoimmunity including RA, multiple sclerosis, and psoriasis. Regarding the detailed structure and signaling pathways of IL-17 and IL-17R we refer to some previous reviews on the subject [22, 23]. Briefly, the IL-17 cytokine family consists of six currently known members (IL-17A–F) and five receptors (IL-17RA–RE) in mammals. IL-17A is the most prominent member of the family and is simply referred to as IL-17 by many authors. IL-17A and IL-17F are closely related, both secreted by Th17 cells and having an amino acid sequence homology of 50%. IL-17A can form homodimers, or heterodimers with IL-17F; both forms are biologically active through the binding of IL-17-RA, although the IL-17A homodimer is more potent [24].

## 4. The Role of IL-17 in Rheumatoid Arthritis (RA)

The human IL-17A was first cloned in 1996 and, first time, it was found to be produced by activated CD45(RO)^+^ memory helper T cells [25]. It was shown in the same study that IL-17A induces the production of IL-6, IL-8, PGE_2_, and G-CSF in a dose-dependent manner in cultures of RA synovial fibroblasts. The IL-17A effect was blocked with anti-IL-17 antibodies [25]. Interestingly, TNF*α* had an additive effect on IL-17-induced secretion of IL-6. Soon after these observations it was found that synovial fluids of RA patients have high IL-17A levels compared to those with osteoarthritis. IL-4 or IL-13 completely inhibited the IL-17 production of* ex vivo* cultured RA synovium tissue [25], whereas exogenous IL-17 increased IL-6 production in synovial tissue cultures. These observations led to the conclusion that through the production of other proinflammatory cytokines, IL-17 has a significant, if not a central, role in the pathogenesis of RA [25].

## 5. IL-17 Regulates Bone Resorption in Human RA

Destruction of the articular cartilage accompanied by the juxta-articular bone resorption and marginal erosions in the bone are prominent histological features of RA [1]. Osteoclasts are large, multinucleated cells responsible for the degradation of bone [26]. It has been shown that certain cytokines play a major role in the differentiation of osteoclasts, although this field is complicated to study because many cytokines have both stimulatory and antagonistic effects on osteoclastogenesis, and their net effect is determined mainly by the specific bone microenvironment [27]. The cytokines promoting osteoclastogenesis act mostly via the RANKL expression, although some proinflammatory cytokines such as IL-1, IL-6, and TNF*α* might be able to induce osteoclastogenesis independently of RANKL. The introduction of TNF*α*-inhibitors along with IL-1 and IL-6 antagonizing therapies further confirmed that these cytokines play a crucial role in bone and cartilage destruction, as these drugs proved to have a major protective effect on bone resorption [28].

IL-17, in particular, is also considered to be osteoclastogenic [29]. In an* in vitro* model of osteoclastogenesis, cocultured murine osteoblasts and hematopoietic cells were treated with IL-17 derived from the synovial fluids of RA patients resulting in an increased IL-17-dependent osteoclastogenesis [30]. Interestingly, this also induces an increased PGE_2_ production in osteoblasts, and the addition of a COX2 inhibitor (e.g., indomethacin) had an inhibitory effect on osteoclast formation [30]. Later, it was shown that IL-17 is involved in increased bone resorption in human RA bone explant cultures and enhanced proteoglycan loss from mouse cartilage [31]. Another research group reported that IL-17 promoted osteoclastogenesis* in vitro* from human CD14^+^ osteoclast precursors acquired from healthy donors through the upregulation of the receptor activator of NF-*κβ* (RANK) [32].

Interestingly, some human *γδ* T cells, capable of producing IL-17 under certain conditions, have been recently described in the peripheral blood [33], and it has been also confirmed earlier that the level of *γδ* T cells in the synovial fluids of RA patients was elevated compared to healthy controls [34]. Even though IL-17 producing *γδ* T cells are known to contribute to the pathogenesis of murine models of RA [35], activated *γδ* T cells seemed to inhibit osteoclastogenesis in an IFN*γ* dependent manner* in vitro*  [36]. IL-17 producing (IL-17^+^) CD4^+^ cells infiltrated the synovium and these CD4^+^IL-17^+^ T cells were detected in close proximity of osteoclasts in the joints of RA patients [37]. The introduction of anti-IL-17 therapeutic antibodies is expected to refine our knowledge on the effects of IL-17 regarding altered bone homeostasis in RA.

## 6. IL-17 in Animal Models of RA

Much of our knowledge about IL-17 is derived from animal models of RA. Several mouse models exist that mimic some or more characteristics of the human disease. Herein, we mention only those few autoimmune models, either spontaneous or inducible, in which IL-17 has been proposed to play a role (summarized in Table 1). We will pay special attention to proteoglycan aggrecan-induced arthritis (PGIA) which is perhaps the most complex available immunological model of RA.

## 7. Th17 Cell Driven Arthritis in SKG Mouse

SKG mice spontaneously develop rheumatoid factor- (RF-) positive autoimmune arthritis, which resembles human RA [77]. This mouse strain harbours a point mutation in the gene encoding the Src homology 2 (SH2) domain of Zeta-chain-associated protein kinase 70 (ZAP-70), a key molecule in T cell receptor signaling [77]. The altered thymic selection resulted in highly self-reactive T cells, which spontaneously differentiated into Th17 cells. It had been shown that IL-17 and IL-6 were essential in the development of arthritis, while, unexpectedly, IFN-*γ* had a protective role (Table 1) [38]. The role of IL-17 was further proven because the treatment with nondepleting anti-CD4 monoclonal antibodies prevented the onset of arthritis in SKG mice by altering T_reg_/Th17 ratio in synovial tissue and draining lymph nodes (Table 1) [39]. Furthermore, treatment with neutralizing anti-IL-17A slightly inhibited the progression of arthritis in SKG mice (Table 1) [40].

## 8. IL-17 in K/BxN Mice and the Serum Transfer Model

K/BxN mice express the transgenic T cell receptor KRN against bovine RNase antigen in nonobese diabetic (NOD) background having the MHC class II allele Ag^7^ (present in the NOD strain) spontaneously developing uniform, severe inflammatory arthritis by the age of 4 weeks. In addition to the spontaneous arthritis, the most evident pathological abnormality is the presence of anti-glucose-6-phosphate isomerase (GPI) antibody production in K/BxN mice [78]. Serum transfer from sick K/BxN mice causes a transient arthritis in a wide range of recipient mice [48, 49].

The importance of IL-17 in K/BxN was investigated by neutralization experiments using monoclonal anti-IL-17 antibodies (Table 1). When 25-day-old K/BxN mice housed in specific pathogen-free environment were treated with anti-IL-17 the onset of arthritis was delayed which was associated with a slower disease progression and reduced ankle thickening (Table 1). These anti-IL-17-treated mice also had lower serum levels of GPI autoantibodies (Table 1) [41]. Th17 cells play an important role in disease induction in K/BxN mice, which was supported by the fact that treatment of mice from birth with neomycin exacerbated arthritis, while treatment with vancomycin or ampicillin inhibited disease progression [41], most likely due to the fact that these latter two antibiotics are known to block Th17 T cell differentiation (Table 1). On the other hand, neomycin targets Gram-negative bacteria, which comprises the majority of gut microbiota, and, Th17 cells are known to mediate host defence against extracellular, especially Gram-negative bacteria (Table 1) [79].

Most recently it was investigated whether IL-17 or IL-17 producing cells play a role in the serum transfer model of arthritis (Table 1). Katayama and colleagues transferred K/BxN serum to IL-17A^−/−^ mice and found that the disease was significantly less severe than when the K/BxN serum was transferred into wild-type mice or mice with severe combined immunodeficiency (SCID) (Table 1) [42]. Interestingly, high IL-17 serum levels were observed in transferred arthritic SCID mice, and it was revealed that neutrophils acted as source of IL-17 in the effector phase of arthritis (Table 1). Moreover, coinjection of wild-type neutrophils with K/BxN serum into IL-17A^−/−^ mice resulted in the exacerbation of the disease (Table 1) [42]. K/BxN serum transfer studies into IL-17RA^−/−^ animals resulted in similar findings: reduced clinical signs of arthritis and decreased expression of various chemokines, proinflammatory cytokines, and matrix metalloproteinases (Table 1) [43].

## 9. IL-17 in the Cytokine Network Regulation in Collagen-Induced Arthritis (CIA)

CIA is perhaps the most widely used inducible autoimmune (systemic) arthritis model, which can be induced by repeated intracutaneous immunization of DBA/1 mice with type II collagen (CII) emulsified with complete Freund's adjuvant.

The role of IL-17 in the development of CIA was first proved by the blockade of endogenous IL-17 with soluble IL-17 receptor protein in immunized DBA/1 mice [31], which resulted in the suppression of arthritis, accompanied by reduced joint damage. In contrast, either systemic or local (intra-articular) adenoviral gene transfer of IL-17 exacerbated CIA. These results were supported by Nakae and colleagues [80] using IL-17 deficient (IL-17^−/−^) mice. Compared to wild-type animals both the incidence and severity of arthritis were markedly reduced and joint histology showed milder inflammation. Collagen-specific IgG2a levels were lower than that of wild-type mice.* In vitro* proliferative response to CII was reduced in IL-17^−/−^ lymph node cell cultures, suggesting a crucial role for IL-17 in the induction of CIA. In another study, mice were immunized after allogeneic IL-17^−/−^ bone marrow transplantation; type II collagen-immunized DBA/1 mice developed significantly less severe arthritis associated with reduced production of proinflammatory cytokines [46].

Both IL-17 producing CD4^+^ Th17 cells [52] and *γδ* T cells [53] were described in CIA. Th17 cells are thought to mediate their effects by stimulating osteoclastogenesis [37], but it remains to be evaluated how V4*γ* ^+^V4*δ* ^+^ *γδ* T cells contribute to the development of CIA via their IL-17 production.

Over the years various IL-17 blocking antibodies were tested in CIA (summarized in Table 1). The severity of the disease was successfully reduced using polyclonal rabbit anti-murine IL-17 antibody [47], single-chain bispecific antibody (scBsAb1/17) against both human IL-1*β* and human IL-17A [48], combination therapies with anti-IL-1*β* and anti-IL-17A antibodies [49] or anti-IL-17 and anti-GM-CSF antibodies [51], or bispecific and neutralizing antibodies (BsAB-1, BsAB-2 and BsAB-3) against both human IL-1*β* and human IL-17A [50].

IL-17 exerts its proinflammatory actions through various pathways (Table 1). IL-17 upregulates other proinflammatory cytokines, such as IL-6 and IL-1*β* [44, 59]. IL-6 induces Th17 differentiation forming a positive feedback loop triggered by IL-17A [54]. Thus, the blockade of IL-6 in the early phase of CIA inhibits Th17 differentiation, hence suppressing disease progression [55]. Bone erosion in CIA is mediated by IL-17 and Th17 cells through the regulation of RANKL-mediated osteoclastogenesis [63, 64].

IL-17 was also found to contribute to angiogenesis, as local overexpression of IL-17 using adenoviral vectors resulted in increased endothelial staining in the ankles of CII-immunized mice compared to controls (Table 1) [44].

IL-17 production or differentiation of IL-17 producing cells is regulated by various cytokines and cells [3]. Intra-articular overexpression of IL-4 using adenoviral vectors resulted in reduced synovial IL-17 mRNA levels and prevented joint damage and bone erosion in CIA by suppressing osteoclastogenesis [57]. Sarkar and colleagues cocultured IL-4-transduced dendritic cells with splenic T cells from CII-immunized mice and found that IL-17 production by T cells was significantly reduced when T cells were harvested during the initiation phase of the disease, but that of T cells obtained during the end phase was not altered [81]. This result is particularly important and interesting because it shows that even during the progression of the disease there could be a shift in the cytokine balance and/or regulation, whose observation was further supported by results from the PGIA model (see Section 10) [82].

A number of cytokines regulate CIA in a network-like fashion (see also Table 1):

(i) IL-12 is an important cytokine in Th1 differentiation by promoting IFN*γ* production. CIA is more severe in mice deficient of IL-12, accompanied by increased IL-17 mRNA levels, suggesting that IL-12 regulates IL-17 production in CIA [58].
(ii) Local expression of IL-27 in the ankles of mice ameliorated CIA by reducing IL-17 levels in the serum and joints of animals [59].
(iii) Monocyte and neutrophil recruitment as well as angiogenesis was inhibited in synovial tissue, and the expression of downstream targets of IL-17, such as IL-1*β*, IL-6, and CCL2, was reduced [59].
(iv) IL-37, a recently discovered member of IL-1 family, is expected to play an immunosuppressive role in CIA via the downregulation of IL-17 and Th17 cell proliferation [60].
(v) NK cell depletion exacerbated experimental arthritis, supporting a possible protective role of NK cells in CIA by inhibiting Th17 differentiation via their IFN*γ* production [61].
(vi) Regulatory B cells control autoimmunity by their IL-10 production by means of promoting T_reg_ differentiation over Th1/Th17 differentiation [62]. IL-10 signaling in T cells is important in ameliorating CIA, as blocking this pathway rendered mice highly susceptible to arthritis via increased IL-17 levels and accumulation of IL-17 producing *γδ* T cells in the joints [63].
(vii) IL-23 proved to be essential in the development of CIA, supported by the fact that IL-23p19^−/−^ mice were resistant to arthritis, with no signs of bone or joint destruction. Results indicated that IL-23 promoted differentiation of IL-17 producing CD4^+^ T cells [58]. In contrast, immunization with peptide-based vaccines targeting the IL-23p19 subunit resulted in suppressed arthritis, but IL-17 mRNA level and T cell populations in the spleen were not altered [64].
(viii) IFN*γ* receptor deficient mice develop exacerbated CIA [65]. Preventive treatment of these mice with anti-IL-17 inhibits CIA, with no signs of bone destruction, neutrophil infiltration, and granulopoiesis. It is supposed that besides inhibiting Th17 differentiation IFN*γ* protects from autoimmunity by inhibiting effector functions of IL-17 [66].

## 10. PGIA at the Border of Th1/Th17 Disease

In genetically susceptible BALB/c mice repeated intraperitoneal immunizations with human cartilage proteoglycan (aggrecan) emulsified with a synthetic adjuvant lead to chronic joint inflammation [83], resembling human RA in both clinical (progressive irreversible cartilage destruction, bone erosion, and ankylosis) and immunological characteristics (T cell-dependent, autoantibody-driven disease) [84].

Originally, PGIA was thought to be completely a Th1-type disease with significant IFN*γ* production [85]. Finnegan and colleagues demonstrated that in contrast to CIA, IFN*γ* deficiency or treatment with anti-IFN*γ* antibody resulted in the amelioration of arthritis. Similarly to CIA, PGIA is also controlled by a network of cytokines (Table 1): arthritis severity is regulated by IL-4 and IL-12 production as shown by testing PGIA in a number of knock-out animals [67].

Later, it was demonstrated that, during the immunization period of PGIA, that is, even before the onset of clinical signs, IL-17 is a prominent proinflammatory cytokine, produced in significant amounts both locally in the peritoneal cavity and systemically in the spleen or lymph nodes of mice (Table 1) [82]. This suggested that both Th1 and Th17 cytokines were involved in the development of PGIA. However, as implicated above (see Section 9), the cytokine balance could shift during the disease progression.

After repeated intraperitoneal immunizations with PG, resident B1 cells were replaced by T cells and conventional B cells in the peritoneum, which may trigger the effector phase of arthritis [82]. B1 cells shift the immune response towards Th1/Th17 direction, while conventional B cells favour T_reg_ induction [75, 86]. Although the number of B1 cells significantly decreased in the peritoneal cavity upon immunization [82], the residual cells seemed to have sufficient capacity to maintain Th1/Th17 polarization in BALB/c mice or, alternatively, their presence is only necessary during the initiation of the disease.

The role of IL-17 in PGIA was further investigated in IL-17 deficient (IL-17^−/−^) mice, in which, surprisingly, the onset and severity of arthritis were equivalent to wild-type animals. Although the expression of IL-1*β* increased significantly in the inflamed joints, IL-6 expression was suppressed. Joint histology and PG-specific T and B cell responses were similar in IL-17^−/−^ and wild-type mice, suggesting that IL-17 was not essential in PGIA (Table 1) [68].

There was a clear contradiction between the earlier mentioned study [82], where significant IL-17 production was detected in PG-immunized mice, and the latter results when IL-17^−/−^ mice were used [68]. The discrepancy between the two observations was solved, when the relation of IL-17 and IFN*γ* was assessed in the same model (PGIA) system (Table 1). In PG-immunized IFN*γ*-deficient mice IL-17 levels were significantly increased [69]. Double knockout (IFN*γ* ^−/−^/IL-17^−/−^) mice were then immunized to investigate the relationship between IFN*γ* and IL-17 in PGIA (Table 1). Compared to wild-type, IFN*γ* ^−/−^, or IL-17^−/−^ mice, severity and onset of arthritis were significantly reduced in the double knockout mice, suggesting that PGIA “became” IL-17-dependent in the absence of IFN*γ*. Cellular infiltration in the synovium and joint destruction were diminished in double knockout IFN*γ* ^−/−^/IL-17^−/−^ mice. The impaired migration of Th17 cells into the inflamed joints might be explained by the reduced synovial expression of CCL20, a ligand of chemokine receptor CCR6 on Th17 cells [69]. Results from IFN*γ* ^−/−^/IL-17^−/−^ mice underline the network-like function of cytokines in PGIA: the absence of IL-17 was compensated by the overproduction of IFN*γ*. This may as well explain why IL-17^−/−^ mice developed PGIA, and, in turn, the overproduction of IL-17 in IFN*γ*-deficient mice can be responsible for the residual disease activity.

## 11. Concluding Remarks about the Experimental Autoimmune Models of RA

There are many animal models of RA, which might represent the heterogeneity of the human disease itself. Above, we reviewed the involvement of IL-17 and IL-17 producing cells in some spontaneous (SKG and K/BxN) and inducible (K/BxN serum transfer, CIA, and PGIA) animal models of RA (summarized in Table 1). Based on the studies available, the autoimmune arthritis observed in SKG, K/BxN serum transfer, and CIA models might be clearly IL-17-dependent disease. In contrast, PGIA might represent a transition between Th1 and Th17 mediated forms of autoimmune arthritis [69].

These results clearly exemplify that in the development of experimental autoimmune arthritis various cytokines and immune cells [61, 62] act in close association, proving the network-like functioning of the immune system. Nevertheless, when working with animal models one must be aware of the characteristics of the chosen model and take into consideration that because of the complex interactions of cytokines genetic modifications might result in diverse compensatory mechanisms.

## 12. IL-17 as a Therapeutic Target in RA

Since IL-17 seems to play a major role in various autoimmune disorders characterized by chronic inflammation, several studies reached the conclusion that antagonizing IL-17 could be beneficial in these pathological conditions [87]. Various IL-17 blocking agents have been developed and are currently being tested in RA (summarized in Table 2), psoriasis, ankylosing spondylitis, and inflammatory bowel diseases, including Secukinumab, Ixekizumab, and Brodalumab [87].

Secukinumab, manufactured by Novartis Pharma AG under the trade name Cosentyx, formerly referred to as AIN457, is a human monoclonal IgG1 that targets IL-17A (Table 2). The safety and efficacy of the drug were first tested in a small number of patients with psoriasis, RA, and chronic noninfectious uveitis and produced clinically relevant response rates of different magnitude in each of the above-mentioned disorders [70]. The drug was approved by the United States Food and Drug Administration (FDA) for the treatment of psoriasis in January 2015 and is currently under investigation in RA (Table 2) and psoriatic arthritis [88].

The results of a phase II double-blind, randomized, placebo-controlled trial about the effectiveness of Secukinumab including 237 RA patients with incomplete responses to previous methotrexate therapy was published in 2013, in which different doses of Secukinumab (25, 75, 150, and 300 mg per month, resp.) were administered subcutaneously for a period of 16 weeks on a monthly basis [71]. Injections of 75, 150, and 300 mg Secukinumab reduced the levels of serum C-reactive protein (CRP) compared to placebo and also decreased the DAS28 (disease activity score 28) of patients, but the primary efficacy endpoint defined by the ACR20 (ACR: American College of Rheumatology) was not achieved (Table 2). The trial was extended to a period of 52 weeks to investigate the long-term effects with 174 of the original 237 patients [72]. The patients with improved CRP levels and DAS28 scores sustained their responses throughout the treatment, and the response of those patients who received 150 mg of Secukinumab was improved through week 52 (ACR50: week 16 = 45%, week 52 = 55%) (Table 2). Mostly mild or moderate adverse effects were found in 64.8% of the patients, and serious adverse effects were detected only in 8.9% of the patients. Based on these results, the authors concluded that 150 mg of monthly Secukinumab treatment can produce clinically relevant response in patients who previously failed to respond to cDMARD or bDMARD treatments [72]. The effectiveness and safety of Secukinumab are now being investigated in two phase III clinical trials (NCT01377012 and NCT01350804).

Ixekizumab is an anti-human IL-17A humanized IgG4 developed by Eli Lilly and Company under the name LY2439821 (Table 2). The first proof-of-concept study consisted of two phases. First, 20 RA patients received 1 intravenous dose of Ixekizumab or placebo and patients were regularly controlled by an 8-week period (Table 2). In the second study, 77 RA patients being already on stable doses of at least one DMARD received one intravenous dose of either Ixekizumab or placebo 5 times with two-week intervals (Table 2). Intravenous Ixekizumab added to oral DMARD therapy improved both DAS28 and the ACR20 scores greater than placebo [73]. The initial study was followed by a phase II clinical trial in which Ixekizumab was given subcutaneously to 260 bDMARD naive patients and to 188 patients with inadequate responses to anti-TNF*α* biologics combined with cDMARD therapies [74]. Using a logistic regression model, in the biologically naive group, a dose-related response rate was detected at week 12 by measuring ACR20 response rates. In patients with inadequate response rates to TNF*α*-blocking agents, Ixekizumab also produced clinically improved ACR20 responses [74]. However, Ixekizumab has not been approved by the FDA to date, but there are ongoing phase III clinical trials in psoriasis (NCT01597245), ankylosing spondylitis (NCT01870284), and psoriatic arthritis (NCT02349295) (Table 2). Recently, no additional studies are performed with RA patients.

Brodalumab is also a novel therapeutic monoclonal antibody that targets the IL-17 system, although, unlike the previously listed agents, it directly binds to the IL-17RA. Brodalumab is a human IgG2 monoclonal antibody developed by Amgen Inc. under the name AMG 827 (Table 2), even though Brodalumab was found to be effective in psoriasis in phase II clinical studies [41, 79], and phase III trials are still ongoing in moderate to severe psoriasis (NCT01708590). The efficacy of Brodalumab in RA was not confirmed by clinical studies (Table 2). The results of a phase I study were published in 2013, in which 40 human subjects with methotrexate resistant moderate-to-severe RA were treated with Brodalumab [75]. Although the drug blocked the IL-17RA in circulating leukocytes, it did not have a clinically significant effect on the response rates as by day 85 (Table 2) [73]. A total of 252 RA patients with inadequate responses to methotrexate were included in a subsequent phase II trial which also failed to find evidence on the clinical efficacy and, therefore, it was concluded that Brodalumab seems to be ineffective in RA (Table 2) [76].

The fact that the effectiveness of biological therapies targeting different proinflammatory cytokines differs from patient to patient seems to support the hypothesis of the heterogeneity of RA in terms of the possible underlying pathomechanisms and responsiveness to certain cytokines contributing to disease development in different RA patients. Moreover, while IL-17 is a relatively new therapeutic target in RA, IL-17, and IL-17R family members show a high variability in the expression in individual patients [10]. Therefore, it is not surprising that the blockade of IL-17A or its receptor with monoclonal antibodies did not lead to complete disease remission so far. As indicated in the above sections, discussing the animal models of RA, IL-17, and Th17 cells is part of a very complex immunopathological network, where targeting one single entity might not be sufficient in suppressing the autoimmune process. Thus, currently it is more likely that IL-17 targeting agents could be used to complement/augment current therapies.

However, it cannot be completely ruled out that, in the future, targeting the IL-17 axis in RA at different levels (Th17 differentiation, receptors, signaling, etc.) will not provide better therapeutical results than the currently available monoclonal antibodies. Moreover, we cannot neglect the complexity of the IL-17 system itself, which consists of 6 members with 5 known receptors, thus there is still place for developing new blocking/modifying agents, which might offer exciting new treatment forms in RA.

## 13. Concluding Remarks

Our knowledge has increased significantly about IL-17 and Th17 cells in the past 20 years. It became clear that this proinflammatory cytokine plays a key role in autoimmunity and more specifically in RA. We have detailed information about the immunological role of IL-17 based on different mouse models of arthritis complemented by some human data. However, as indicated above, care should be taken when analysing data derived from knock-out models and other genetically engineered mice, because the network-like function of the immune system might lead to unexpected compensatory mechanisms which could significantly alter the results. Nevertheless, our current understanding about IL-17 in RA (and new potential treatment directions) would not exist without the data from animal models. Therapeutic trials aiming to suppress IL-17 might provide some new treatments supplementing or replacing currently existing biological therapies in RA.

## Figures and Tables

**Table 1:** Summary of the most important data about IL-17 in animal models of RA.

Model	Experiment	Effect	Reference
SKG	IL-17^−/−^ mice	Inhibited arthritis	[38]
	IL-6^−/−^ mice	Inhibited arthritis	[38]
	IFN-*γ* ^−/−^	Exacerbated arthritis	[38]
	a-CD4	Prevented arthritis (altering T_reg_/Th17 ratio)	[39]
	a-IL-17A	Inhibited arthritis progression	[40]

K/BxN	a-IL-17	Slower disease progression	[41]
	Neomycin	Exacerbated arthritis (Th17 differentiation)	[41]

K/BxN serum	IL-17A^−/−^ mice	Less severe disease	[42]
	IL-17RA^−/−^ mice	Reduced disease severity	[43]
	Neutrophils	Source of IL-17 in effector phase	[42]

CIA	Soluble IL-17R	Suppression of arthritis	[31]
	Ad-IL-17	Exacerbation of arthritis	[31]
		Angiogenesis	[44]
	IL-17^−/−^ mice	Suppression of arthritis	[45]
	IL-17^−/−^ BM transfer	Suppression of arthritis	[46]
	a-IL-17 serum	Suppression	[47]
	a-IL-1*β* + a-IL-17A (sc, bs)	Suppression	[48]
	a-IL-1*β* or a-IL-17A (c)	Suppression	[49]
	a-IL-1*β* + a-IL-17A (bs, n)	Suppression	[50]
	a-IL-17 a-GM-CSF (c)	Suppression	[51]
	CD4^+^ Th17 cells	Stimulation of osteoclastogenesis	[37, 52]
	V4*γ* ^+^V4*δ* ^+^ *γδ* T cells	IL-17 production	[53]
	IL-6	Upregulated by IL-17	[47]
		Positive feedback loop triggered by IL-17A	[54]
		Induces Th17 differentiation	[55]
	IL-1*β*	Upregulated by IL-17	[52]
	RANK, RANKL	Expression induced by Th17 cells	[56]
	Ad-IL-4	IL-17 mRNA levels ↓	
		Prevention of osteoclastogenesis	[57]
	IL-12p35^−/−^ mice	More severe arthritis	
		IL-17 mRNA levels ↑	[58]
	Ad-IL-27	Suppressed arthritis	
		IL-17 levels in joint and serum ↓	
		IL-1*β*, IL-6, and CCL2 expression ↓	[59]
	Ad-IL-37	Downregulates IL-17	
		Inhibits Th17 proliferation	[60]
	NK cell depletion	Exacerbation of arthritis	[61]
	B_reg_ cells	Suppression of arthritis (T_reg_ differentiation ↑)	[62]
	IL-10R Tg mice	Increased susceptibility of arthritis (IL-17 ↑)	
		IL-17 producing *γδ* T cells accumulate in joint	[63]
	IL-23p19^−/−^ mice	Inhibition of arthritis (Th17 differentiation ↓)	[58]
	IL-23p19 vaccine	Suppressed arthritis	[64]
	IFN-*γ*R^−/−^ mice	Exacerbated arthritis	[65]
	IFN-*γ*R^−/−^ mice + a-IL-17	Inhibited arthritis	[66]

PGIA	Initiation phase	Local and systemic IL-17 production	[67]
	IL-17^−/−^ mice	Severity and onset similar to WT	[68]
		IL-1*β*↑ IL-6 expression ↓	
	IFN-*γ* ^−/−^ mice	Amelioration of arthritis	[69]
		IL-17 levels ↑	
	IFN*γ*/IL-17^−/−^ mice	Suppressed arthritis	[69]
		Reduced joint damage and cellular infiltration	

sc: single-chain; bs: bispecific; n: neutralizing; c: combination therapy; Ad: adenoviral; Tg: transgenic; BM: bone marrow.

**Table 2:** Summary of the most important therapeutical trials targeting IL-17 in RA.

Name	Molecular target	Phase	Status	Patients	Weeks	Efficacy	Reference
Secukinumab	IL-17A	I	Completed	52	6	Supported	[70]
		II	Completed	237	16	Supported	[71]
		II	Completed	174	52	Supported	[72]
		III	Ongoing	n/a	n/a	n/a	NCT01377012
		III	Ongoing	n/a	n/a	n/a	NCT01350804

Ixekizumab	IL-17A	I	Completed	97	10	Supported	[73]
		II	Completed	448	12	Supported	[74]

Brodalumab	IL-17RA	I	Completed	40	48	Not confirmed	[75]
		II	Completed	252	12	Not confirmed	[76]

n/a: no information available.

## Abbreviations

ACR: American College of Rheumatology
bDMARD: Biological disease-modifying antirheumatic drug
CII: Type II collagen
CCL20: Chemokine (C-C motif) ligand 20
CCR6: C-C chemokine receptor type 6
cDMARD: Conventional disease-modifying antirheumatic drug
CIA: Collagen-induced arthritis
COX2: Cyclooxygenase-2
CRP: C-reactive protein
CTLA-8: Cytotoxic T-lymphocyte-associated antigen 8
DAS28: Disease activity score 28
DMARD: Disease-modifying antirheumatic drug
FDA: United States Food and Drug Administration
G-CSF: Granulocyte colony-stimulating factor
GM-CSF: Granulocyte macrophage colony-stimulating factor
GPI: Glucose-6-phosphate isomerase
HSV13: Herpesvirus Saimiri 13
IFN*γ*: Interferon gamma
NF-*κβ*: Nuclear factor kappa-light-chain-enhancer of activated B cells
PGE_2_: Prostaglandin E2
PGIA: Proteoglycan aggrecan-induced arthritis
RA: Rheumatoid arthritis
RANK: Receptor activator of nuclear factor kappa-B
RANKL: Receptor activator of nuclear factor kappa-B ligand
RF: Rheumatoid factor
ROR*γ*t: RAR-related orphan receptor gamma t
SCID: Severe combined immunodeficiency
SH2: Src Homology 2
TNF*α*: Tumor necrosis factor alpha
ZAP-70: Zeta-chain-associated protein kinase 70.
